# An Account of the Successful Termination of a Case Attended with Symptoms of Phthisis Pulmonalis; with Remarks on the Treatment of That Disease

**Published:** 1788

**Authors:** William May

**Affiliations:** Physician at Truro in Cornwall


III. *An Account of the ſucceſsful Termination of a Caſe attended with Symptoms of Phthiſis Pulmonalis; with Remarks on the Treatment of that Diſeaſe. By* William May, *M. D. Phyſician at Truro in Cornwall.*

IN the month of December, 1786, I was deſired to ſee a young woman, who was ſaid to to be in an advanced ftage of pulmonary confumption. She was about eighteen years old, had not menftruated, and poffeffed that narrow conformation of cheft, with high fhoulders, a long neck, fine fkin, and white teeth, together with the circumfcribed rednefs of the cheeks, and other general appearances indicating a predifpofition to phthifical affection, and was born of fcrophulous parents.

I found her labouring under every fymptom which can be fuppofed neceffary to characterize a true phthifis pulmonalis; but, left the propriety of this conclufion fhould be doubted, I will here briefly enumerate the particular circumftances of her cafe with as much accuracy as an imperfect recollection of the exact feries in which they happened will allow.

About eight or ten weeks before I faw her fhe was firft feized with a cough, which, in the ordinary way, was fuppofed to be merely catarrhous: it was without expectoration, extremely urgent, and from time to time accompanied with flight pains affecting the thorax, but confined to no particular part of it. She had alfo, at that period of the difeafe, complained occafionally of irregular rigours, followed by heat and flufhing of the face.

After a few weeks paſſed away in this manner, the ſymptoms, by ſlow degrees, growing more and more troubleſome, a frothy mucus was expectorated, which was ſometimes tinged with blood. In a ſhort time this hæmorrhage from the lungs became more conſiderable, and recurred pretty regularly at the ſtated period of four or five days. It was conſtantly preceded by the ſymptoms which Dr. Cullen has taken notice of in his noſological character of hæmoptyſis *, viz. "genarum rubor; moleſtiæ aut doloris, "& aliquando caloris, in pectore ſenſus; dyſ-"pnoea; titillatio faucium." Theſe ſymptoms, produced by the hæmorrhagic effort, were always relieved by the ceaſing of the hæmorrhage, and in the intervals between theſe attacks the ſame kind of mucous matter as has been deſcribed above continued to be expectorated. At length the matter expectorated increaſed conſiderably in quantity, and put on an evident appearance of purulency, and the ſymptoms of pyrexia became more ſtrongly marked.

It was at this period of the diſeaſe I firſt viſited the patient. She was then in a ſtate of extreme debility, and was exceedingly ema-

* Synopſ. Noſol. Method. 8vo. Edin. 1785. Tom. II. p. 156.

ciated.

ciated. The noon and evening exacerbations of true hectic fever, with profuse night sweats, recurred in a very regular succession. The belly was sometimes costive, and frequently affected with profusely colliquative discharges, which seemed to lessen, while they lasted, the discharge by the skin: indeed I never saw the consent between the surface of the body and intestines so clearly pointed out as in this case, in which these different conditions regularly alternated with each other. The pulse was irregular in point of frequency, but invariably above 110: sometimes, especially before the hæmorrhage, full and hard, but at other times small and extremely weak. The matter expectorated, which was now in considerable quantity, submitted to the more common criteria, as well as to those recommended by Dr. Brugmans * and the late ingenious Mr. Charles Darwin, appeared very satisfactorily, and beyond all doubt, to be of a purulent nature. Her nights were restless, and anxious, her breathing laborious and painful, and, if kept out of bed but a few hours, her legs became œdematous. She had the pearly

* Dissertatio Inauguralis Gotting. edit. auct. D. Brugmans, Botanices Leidæ Professore, de Puogenia, 1783.

whiteness whitenefs of the tunica adnata of the eye, the adunque incurvated form of the nails, with the defluxio capillorum which Sydenham, Dr. Cullen, and all other writers, have confidered as certain diagnoftics with refpect to the difeafe.

Under thefe circumftances, contrary to the more general practice in phthifical cafes, of relying on antiphlogiftics, demulcents, and expectorants, which I confider not merely as an inefficacious, but as a dangerous method of treatment, the indication of cure upon which I proceeded was to remove the hectic paroxyfms by obviating the debility of the fyftem upon which they depend, and which, in my opinion, is to be confidered as the fole proximate caufe of this formidable difeafe *.

The means adopted for this purpofe were, firft, fmall dofes of thebaic tincture exhibited at night and in the morning, and in the mean time a diet of the moft nutritious kind was di-

* This opinion of the proximate caufe of phthifis is not a fingular one. Dr. Kentifh, in his inaugural Differtation on this fubject, printed at Edinburgh in 1784, has the following words : — " Si caufa proxima ea fit qua fublata tollitur mor-" bus, ex caufarum remotarum jufta contemplatione hujus " morbi caufam proximam exiftere debilitatem non poffumus " non judicare."

rected.

rected. Soups of all ſorts, and, if the appetite called for it, even ſolid animal food, with liberal portions of wine, were allowed her. For her common drink ſhe was ordered to uſe porter, or brandy and water. In the ſpace of about a week an emetic of ipecacuanha was exhibited, and afterwards the bark in ſubſtance.

This method was perſiſted in with evident advantages for ſome time, with ſuch little variations only of regimen or medicines as either the occurrence of anomalous ſymptoms or the ſtate of her appetite required. It muſt be remarked, however, that ſoon after the exhibition of the bark, the patient began to feel a pain at the ſtomach, accompanied with nauſea. To obviate theſe complaints, the uſe of the bark was ſuſpended, and another emetic adminiſtered, by the operation of which a quantity of palpable powder of bark was rejected, made into a firm maſs with a glutinous viſcid matter in the ſtomach, the evacuation of which entirely removed the troubleſome ſymptoms before mentioned.

Among the articles of diet which ſhe was moſt in the habit of uſing muſt be mentioned eggs and oyſters; the latter of which ſhe deſired with much avidity, and ate in large quantities tities either raw or roaſted, with pepper and other condiments.

The attacks of hæmoptyſis recurred with leſs frequency and violence, and at laſt diſappeared altogether. Still, however, the expectoration of pus, with febrile acceſſions, and much debility, though all in an evidently leſs violent degree, continued to harraſs the patient, who, notwithſtanding ſhe had been now three months confined, bore her illneſs with wonderful reſignation and fortitude. Every part of the tonic plan of treatment was continued, and, in addition to what has been already related, the exerciſe of ſwinging was directed, which was performed by means of a chair ſuſpended by cords near the bedſide of the patient, when her ſtrength would not allow her being taken out of the room in which ſhe had been confined. This was uſed twice a day, for the ſpace of a quarter of an hour at a time, and it never failed to produce a diminution of the frequency of the pulſe, and an alleviation of that general ſenſe of oppreſſion under which ſhe conſtantly laboured.

In the courſe of the diſeaſe the emetics had been repeated with evident advantage, and the doſes of thebaic tincture increaſed to forty or fifty drops three times a day: the bark was also given in a liberal quantity. In short, the strengthening plan of treatment, which was adopted in the most extensive meaning of the term, and most sedulously practiced, produced, in the end, the most flattering success. The expectoration lessened by degrees, and at length, with the febrile symptoms, totally disappeared. The patient grew stronger as these left her, her appetite became excellent, the colliquative discharges disappeared, and, with the assistance of horse exercise, she at last recovered entirely, and is now in an excellent state of health.

REMARKS.

The case I have just recited suggests some obvious remarks, and I imagine the happy event of a method of cure so very opposite to the ordinary mode of treating consumptive cases is calculated to afford considerable information and improvement. If it should be objected to this opinion, that from an individual case we are not licensed to make general conclusions, it may be answered, that this is by no means a solitary instance, and that even in the writings of Celsus we may find sanction for the practice here recommended. — "Cibi vero," says this elegant and experienced author, "esse debent "ex his qui facile concoquuntur, maximeque "alunt; ergo vini quoque necessarius usus "est *."— Had Celsus known the virtues of opium and the Peruvian bark, who can doubt but that, with the ideas of this disease, which the opinion quoted above necessarily argues, he would have laid particular stress also on the use of these excellent remedies.

The practice of bleeding, and every part of the antiphlogistic plan of treatment, has so generally obtained, that it is scarcely necessary for me to take notice of the common opinions with respect to the nature of this formidable complaint, which must have given rise to this practice; nor would it be decent for me to endeavour to point out its pernicious tendency. The frequent and almost uniformly unsuccessful termination of diseases of this kind, under the ordinary methods of treating them, I should imagine would alone have been sufficient to determine that the practice was erroneous, and, independent of all theoretical disquisition, to have induced practitioners to try some opposite methods, however unsupported by hypothetical rea-

* De Medicin. Lib. iii. cap. 22.

soning,

ſoning, or inconſiſtent with the moſt common theoretical notions about the diſeaſe in queſtion. But when it is conſidered that the moſt rational ideas which can be collected from all pathological writers on the ſubject, and the moſt common opinions which obtain reſpecting it, not only favour, but lead directly to, the tonic plan of treatment, and no other, and that the uſe of the oppoſite methods is directly incompatible with theſe opinions, it appears truly wonderful that the former ſhould have ever been neglected, or the latter method ever have been adopted. The limits preſcribed to me by this method of publication will not allow me to go at large into the reaſoning I might adopt in ſupport of the opinion, that there can exiſt no caſe of idiopathic phthiſis in which bleeding, or any part of the antiphlogiſtic regimen, muſt not be improper. Dr. Cullen, whoſe ſkill and experience no one will doubt, ſeems to have ſet this matter, in my opinion, beyond all controverſy: and though it is a truth which I cannot deny, that this venerable and moſt reſpectable Profeſſor, when treating of the diſeaſe from the chair, in the Univerſity to which he does ſo much honour, ſeems to favour, under particular circumſtances, the uſe of the lancet, yet I maintain that ſuch practice is in direct opposition to the principles he has laid down in his publications. This must be obvious on the most superficial observation; witness the character of the disease given in his Nosology, the principal parts of which are; "Corporis emaciatio & debilitas, cum expec- "toratione purulenta *." — If this is true, and it is a fact too notorious to admit of the smallest doubt, can bleeding and the antiphlogistic regimen make any part of a rational indication of cure? It is repugnant to common sense to suppose it.—But, say the advocates of the extenuating plan, however appearances may indicate the contrary, there is certainly present in all consumptive cases an inflammatory diathesis, which must be obviated by antiphlogistic means. Let us compare this inflammatory condition of the system, so properly described by Dr. Cullen in his First Lines of the Practice of Physic, with the character of this disease already quoted from his Nosology.—"I define the phlogistic diathe- "sis," says he, "to be an increase of tone and "contractility of the whole arterial system." — Is there then the least analogy between an emaciation, ciation, with debility of the body, and this ſtate of tone and vigour of the ſyſtem?

Waving, however, farther arguments on the theory of the diſeaſe, I ſhall return to the more immediate purpoſe of this publication, viz. the ſtatement of ſome facts calculated to eſtabliſh the neceſſity of a cordial and nutritious method of treatment in caſes of pulmonary conſumption.

Dr. Kentiſh, in his valuable Diſſertation on this ſubject, already referred to, has ſtrongly recommended tonic remedies, and a nutritious diet, and, among other inſtances of their good effects, has related the following remarkable caſe:—"Unus ex amicis meis, Raius Beckwith, "quum febre hectica, tuſſi violenta, exſcrea-"tione purulenta, ſudoribus colliquativis diu "laboraſſet; *diæta parca lactea ſine fructu*, tan-"dem, contra medici conſilium, victu ple-"niore, oſtraeis, Falerno, & cereviſia, uſus "eſt; ſymptomata maligna diſparuerunt, feli-"citerque convaleſcebat."

Dr. Mudge alſo, in a Diſſertation on the catarrhous Cough, publiſhed ſome years ſince, has mentioned an inſtance of complete cure of phthiſis, which deſerves to be taken particular notice of on many accounts. — A man was admitted into into St. Thomas's Hoſpital with every ſymptom of pulmonary conſumption. He became a patient of Sir Edward Wilmot's, and was ſubjected to the common plan of treatment in ſuch caſes. The man growing worſe, all hope of his recovery was relinquiſhed, and the nurſes ſeem to have been directed to make the little remains of his life as comfortable as poſſible, by adminiſtering ſome wine to ſupport his exhauſted ſtrength, and opiates to alleviate the cough which harraſſed him. Under this alteration of method the patient, to the aſtoniſhment of all who ſaw him, grew better, and, after a long ſtate of convaleſcence, was at laſt diſcharged from the hoſpital perfectly cured. This caſe ſtrongly corroborates the opinion I have offered with reſpect to the treatment of conſumptive caſes; and, farther, it furniſhes us with a fact of conſiderable importance in the hiſtory of this alarming malady, viz. that it is not incurable: a fact which we cannot take too much pains to aſcertain, eſtabliſh, and inculcate. An elegant writer has very juſtly obſerved in his publication on the duties and offices of Phyſicians, that "to pronounce a diſeaſe incurable is often to make it "ſo;" and cautions practitioners againſt making a gloomy prognoſtic, which can only, by inducing inducing anxiety and defpondency in the patient, aggravate the difeafe, without the poffibility of producing a fingle advantage, except the trifling one of faving his own credit with refpect to the event: but this can always be effected in a manner equally fafe, and infinitely more proper.

Having carried thefe general remarks as far as it may be allowable in this place, I fhall make a few additional obfervations on the cafe which has been the more immediate fubject of this paper. I imagine it will be obvious that the hæmoptyfis which preceded it was the *hæmoptyfis ex tuberculo pulmonum* of Sauvages, or that which Dr. Cullen has defcribed under his third fpecies: viz. "Hæmoptyfis phthifica, poft tuffim cum "macie & debilitate diuturnam *." Confequently no doubt can exift of the cafe here recorded having been a true phthifis pulmonalis, which, if not an idiopathic difeafe, (and I am not clear that it is fo in any cafe) was very evidently the confequence of an original fcrophulous diathefis.

The effects which the Peruvian bark produced, as it was firft adminiftered in fubftance,

* Synopfis Nofol. Method. Tom. II. p. 157.

deferve deferve to be taken notice of. The accumulation of the powder in the ftomach has not unfrequently happened under a debilitated ftate of the digeftive organs, and has been the caufe of great uneafinefs in all the inftances I have heard of. Owing to this circumftance, emetics and cathartics have generally been recommended antecedent to the ufe of the bark; but as it often happens that the condition of perfons for whom the bark is neceffary is fuch as will not admit of vomiting or purging with fafety, in order to obviate this difagreeable effect, I would advife the cold infufion of this medicine with calcareous earth, as defcribed and recommended by Dr. Skeete. This preparation was ufed in the progrefs of the difeafe with manifeft advantage, and in a number of other cafes I have feen as good effects produced by the bark adminiftered in this form as could have been expected from the fubftance itfelf.

Vomiting, however inconfonant it may appear with the reafoning I have attempted, is certainly to be confidered as a ufeful remedy in the treatment of pulmonary cafes. Full vomiting, however, though it muft eventually debilitate, does, by a primary operation, give tone, vigour, and excitement, both to the ftomach and the ſyſtem at large: if, therefore, the excitement which it occaſions is ſuſtained by a judicious exhibition of ſtimulants and tonics immediately upon the ceaſing of the emetic operation, how efficacious repeated vomits muſt be in theſe, and all other caſes of debility, muſt be obvious to every man. In phthiſical affections eſpecially they are calculated to be of the utmoſt utility as expectorants, and, in my opinion, there is ſcarcely any other medicine to which this term can with propriety be applied. By the action of the diaphragm and intercoſtal and pectoral muſcles, as in the effort of vomiting, together with the compreſſion of the air in the cavity of the lungs by the ſhutting of the glottis, which neceſſarily happens, expectoration is more effectually promoted than by any other means I am acquainted with.

Although in the caſe of this patient, from the fulneſs and hardneſs of the pulſe, with the fluſhing of the face, preceding each hæmorrhage from the lungs, bleeding may appear to have been indicated, I am perſuaded that neither in this, nor any other inſtance of the kind, it would have been proper to uſe the lancet. It is true that drawing blood may, by leſſening the plethora of the lungs, give a ſpecies of temporary relief; but it is indeed a moſt fallacious ſpecies,

 and and can never fail to aggravate, by an ultimate and unavoidable operation, the very ſymptom it ſeems calculated to remove.

If, from any thing I have here delivered, it ſhould appear that the ordinary method of treating phthiſical affections is erroneous, and an oppoſite plan, which, in the above inſtances, has been accompanied with ſuch excellent effects, ſhould obtain a fair trial, and be found extenſively uſeful, I ſhall feel myſelf happy in the idea of having contributed ſomething towards the improvement of medical ſcience, and the benefit of ſociety at large. Independently of all hypotheſis on the ſubject, the practice I have here recommended has the beſt arguments in its favour; namely, the conſtant failure of the oppoſite practice, and the ſucceſsful termination of a few, at leaſt, of thoſe caſes in which it has hitherto been adopted. Let it have a candid trial, and ſtand or fall by the teſt of repeated experience. No one will accuſe a practitioner of temerity for proceeding on theſe grounds: he will not add to the opprobrium which this diſeaſe has brought upon phyſic, even ſhould the event of his trials be generally unfortunate; neither ought he to conclude, though this ſhould happen to be the caſe, that the method of cure he has adopted is, therefore, wrong, but to attribute his failure lure either to his own improper management of the remedies employed, or to the invincible nature of the diſeaſe. With reſpect to the latter, however, I agree with the late Dr. Gregory, that no diſeaſe ought to be conſidered as incurable. — This declaration cannot bring upon the profeſſion the charge of arrogance; becauſe we muſt, at the ſame time, confeſs that there are diſeaſes which we do not know how to cure. I am extremely willing to believe that the phthiſis pulmonalis is not among this number, and am happy to be able to corroborate this opinion by a quotation from our Engliſh Hippocrates, (whoſe authority is ſecond to that of no man among the ancients or moderns) expreſsly to this purpoſe—" Quantumcunque exitialis phthi- " ſis et ſit et audiat, utpote qua intereunt duo fere " trientes eorum quos morbi chronici jugulant; " hoc tamen ſancte affero, quod neque mercu " rius in lue venerea, neque cortex Peruvianus " in intermittentibus efficaciores extent, quam in " phthiſi curanda exercitium jam laudatum *."

*Truro,*

July 19, 1788.

* Vide Sydenhami opera, 8vo. Londini, 1705. p. 383 and 384, where he is ſpeaking of the efficacy of riding on horſeback in the cure of phthiſis.

